# One Hundred Consecutive Cases of Skin Disease

**Published:** 1891-01

**Authors:** M. B. Hutchins

**Affiliations:** Atlanta, Ga.; Lecturer on Diseases of the Skin, Atlanta Medical College


### ONE HUNDRED CONSECUTIVE CASES OF SKIN DISEASE.

BY M. B. HUTCHINS, M. D.,
Lecturer on Diseases of the Skin, Atlanta Medical College.

The cases which I shall attempt to describe, in something of an analytical manner, comprise the first one hundred recorded in my practice in Atlanta. I hope through this and following brief papers to bring something of interest to the eyes of the general practitioner, who is too busy to give his time to the extensive literature of dermatology. When it is considered necessary, reasons for the diagnosis will be given; and the treatment of individual cases or groups of cases detailed as far as practicable. There shall be no attempt at selection of favorable cases, and when the result is not given it will be because I was not able to learn it—many cases not taking the trouble to report.

I have made it a rule to employ only such prescriptions as I thought most suitable, and have made no attempt at experiment with a variety of remedial measures. Some patients were able to present two skin diseases, thus making the total of diseases more than that of patients.

Of all varieties of eczema there were thirty-nine cases. Thirteen of these were of the seborrhoic type. All varieties of acne comprised fifteen cases. Of syphilis, with skin manifestations, there were eight cases. Of trichophytosis, or various forms of ringworm, six cases. There were five cases of impetigo contagiosa, "impetigo simp." included according to Crocket. Of psoriasis, epithelioma, ulcus varicosus, pompholyx, and urticaria, each two cases. Tinea versicolor (or chromophytosis), scleroderma, sycosis, nævus vasculosus, carcinoma cutis (probable), erythema multiforme, molluscum epitheliale, "cinders in skin," tuberculosis cutis papillomatosa, pruritus ani, rosacea simplex, "rhus" dermatitis, alopecia areata, algesia cutis, hypertrophia cutis labii inf., papilloma (or verucca) cutis, xeroderma pigmentosum (probable), and keratosis pilaris, of each one case.

Of the twenty-six ordinary eczemas, three affected the face alone ; three the palms, fingers or hands ; four were extensive enough to be called "general ;" two affected only the lower lip ; two on scrotum, one of these with also a few eczematous patches on scalp ; one of eyelids, in strumous child ; one of foot, with slight scaling in ears ; the remainder of the twenty-six being so distributed as to render needless any attempt at specifying the exact position. As to type, two were classified as erythematous ; one, on scabietic patient, as "post scabies" (quite characteristic) ; one vesicular (general) ; result of application of iodoform, two ; "trade eczema," one ; chronic squamous, two ; papular, result of dye-poisoning, one ; pustular, one ; papulo-vesicular (face), one ; remainder of mixed type.

Erythematous type.—First, a boy of two and one-half years, nearly entire skin surface at one time affected ; skin reddened, slightly thickened and scaling ; here and there, small denuded lines or points from which there was slight oozing. Was improved by the use of diachylon ointment, prescribed by another physician. When I saw the case, in consultation, I added to the above, ac. salicyl. gr. x. and amylum gr. xl., to the ounce. All, save scrotum, practically recovered under this treatment, and that received permanent relief, so far as I learned, from the painting on of

R. Picis liquidæ, ʒii.

Ether. sulph.,

Spir. vin. rect., aa ℥ii.

M.

Several relapses occurred on general skin, I learned, after I ceased treating the case. Child had meanwhile had measles and whooping-cough, added to which was the probable suspicion that the diet was unsatisfactory.

The second case of erythematous type was that of a laborer, aged 45. Affected face, neck, arms, hands (skin here thick and severely fissured), scrotum and popliteal spaces. Skin livid red, scaly, hot and itchy. "Oozing points," as in first case. This and former attacks began with redness and swelling about the orbits, extending over face. Urine scant and red. Ordered :

R. Potass. citrat., ʒiv.

T. nucis vom., ʒiss.

T. cinchon. comp., ℥i.

Aquæ ad., ℥iii.

M. Sig.—ʒi, after meals.

R. Ac. sal., ʒiss.

Potass. carb., ʒi.

Ungt. zn. ox., ℥vi.

M. Sig.—Keep well applied and change b. i. d.

Result in this case impossible to learn.

Eczema following scabies, case that of a young man of 18 years. On left elbow a patch of thickened skin covered with dried serous discharge. At anterior border of each axilla redness and minute papules. On glans penis two or three large papules, thickened, "scratched-looking," and covered with dried serum. Similar lesions on scrotum. Redness on inner sides of thighs, with presence of minute papules. Two bed-fellows similarly affected in previous four months, itching marked. Got well under painting with:

R. Picis liquidæ,
Spir. camphoræ, aa ℥i.
Ether sulph.,
Spir. vin. rect., aa ℥iii.

M.

In two cases, both women; one with "varicose," and the other with "specific," ulcer of leg; iodoform, plain, had been applied. Not only were the ulcers not benefited, but the surrounding skin had become the seat of an "iodoform eczema," or dermatitis. Skin was reddened, scaly, thickened, slightly fissured and hot. Removal of iodoform and the application of a simple protective and soothing ointment, as diachylon, or oxide of zinc ointment with a little salicylic acid, caused the rapid disappearance of the inflammation.

Of eczema of the lower lip alone, there were two cases. The first in a young man of twenty-two, hardware salesman. Began as labial herpes over a year previously, gradually becoming red, infiltrated (on carmine border) fissured and superficially excoriated with crusting. Patient was in a low state of health and did not improve until the fall weather came.

R. Liq. potass. ars.,
Tinct. nucis vom., aa ʒii.
Tinct. cinchon. comp., ℥ii.
Aquæ ad, ℥iv.

M. Sig.—ʒi in water after meals.

During the day a three per cent. lotion of ichthyol was employed, and at night a four per cent. ointment of same. At first there was some improvement, but lip afterwards returned to former condition; and there was evidence of slight toxic effect from the arsenic. Arsenic was finally discontinued and the following local application made:

R. Pic. liq., ʒii.

Glycerin, ʒi.

Ether sulph.,

Sp. vin. rect., aa ℥i.

M. Sig.—Paint on and let dry.

At night applications were made of very hot water. Lip got down to slight redness, with here and there a thickened point, and remained practically in that state; patient for some reason not taking advice to go away, rest and improve his general health. Cure seemed impossible without this.

Second case that of a negro, age twenty-five, laborer; disease slight, symptoms showing a modified type of first case. "Dyspeptic stomatitis" also present. Well in a month, under the same prescription as above.

(*Eczemas continued next issue.*)

No. 16½ Whitehall St.

